# Acute Myeloid Leukemia With *CEBPA* Mutations: Current Progress and Future Directions

**DOI:** 10.3389/fonc.2022.806137

**Published:** 2022-02-01

**Authors:** Long Su, Yuan-Yuan Shi, Zeng-Yan Liu, Su-Jun Gao

**Affiliations:** ^1^Department of Hematology, The First Hospital of Jilin University, Changchun, China; ^2^State Key Laboratory of Experimental Hematology, National Clinical Research Center for Blood Diseases, Institute of Hematology & Blood Diseases Hospital, Chinese Academy of Medical Sciences & Peking Union Medical College, Tianjin, China; ^3^Department of Hematology, Binzhou Medical University Hospital, Binzhou, China

**Keywords:** acute myeloid leukemia, *CEBPA* mutations, subsets, prognosis, treatment

## Abstract

Mutations in *CCAAT enhancer binding protein A gene* (*CEBPA*) are one of the common genetic alterations in acute myeloid leukemia (AML). Recently, the emergence of new evidence makes it necessary to reconsider the subsets and treatment of AML patients with *CEBPA* mutations. This review will summarize the history of research progress of *CEBPA* mutations in AML, the heterogeneities of AML with *CEBPA* double mutations (*CEBPA*^dm^), and two special subtypes of *CEBPA* mutated AML. We will discuss the treatment of AML with *CEBPA* mutations as well, and finally propose a new algorithm for the treatment of these patients, including both familial and sporadic *CEBPA* mutated AML patients. This review may be beneficial for further investigation and optimizing clinical management of AML patients with *CEBPA* mutations.

## Introduction

CCAAT enhancer binding protein alpha (CEBPα) is a crucial transcription factor for the differentiation of granulocytes, which also plays a critical role in regulating glucose metabolism (1). CEBPα is encoded by the *CEBPA* gene located in chromosome 19 of human, which contains two transactivation domains (TAD) in the N-terminal and one basic leucine zipper region (bZIP) in the C-terminal. *CEBPA* mutations are one of the most frequent genetic lesions in patients with acute myeloid leukemia (AML). Although mutations of *CEBPA* gene can occur across the whole gene, they cluster in two main hotspots: N-terminal frame-shift insertions/deletions and/or C-terminal in-frame insertions/deletions. Mutations in the N-terminal result in the production of a truncated protein p30, which has a dominant negative effect over the full-length p42 protein, while mutations in the C-terminal will disrupt the binding of CEBPα to DNA or dimerization (2). *CEBPA* mutations include those locate in one terminal (*CEBPA* single mutation; *CEBPA*^sm^) and those that occur in both N- and C-terminals (*CEBPA* double mutations; *CEBPA*^dm^). Although *CEBPA* mutations are widely investigated in numerous studies and several reviews have already been published to discuss their molecular mechanisms and clinical relevance (3–7), newly emerging evidence makes it necessary to reconsider the pathogenesis, subsets, and treatment choice of AML with *CEBPA* mutations. The aim of this perspective review is to summarize the latest findings in this field and propose a new treatment algorithm based on the available evidence.

## Key Research Progress of *CEBPA* Mutations in AML

**Figure 1:**
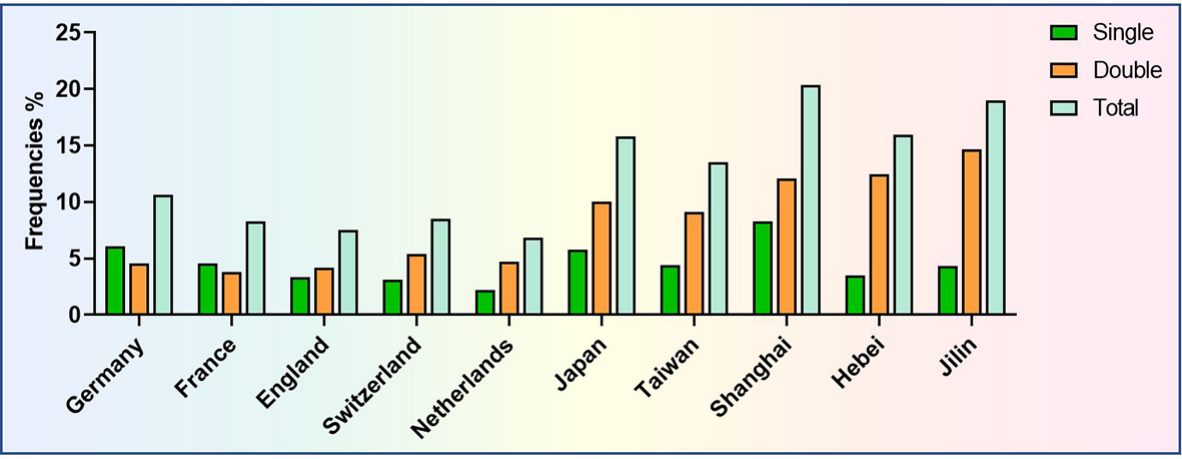
Frequencies of *CEBPA* mutations in AML patients from different countries or different regions of China.

The frequency of *CEBPA* mutations in AML is 6.86%–20.33%, and a higher incidence rate is observed in AML patients from Asia compared to that in Western countries. Moreover, the frequencies of *CEBPA*^sm^ and *CEBPA*^dm^ are similar in AML patients from Caucasian populations, but more patients present with *CEBPA*^dm^ in Asian populations (2, 6, 8–13) (**Figure 1**).

**Figure 2:**
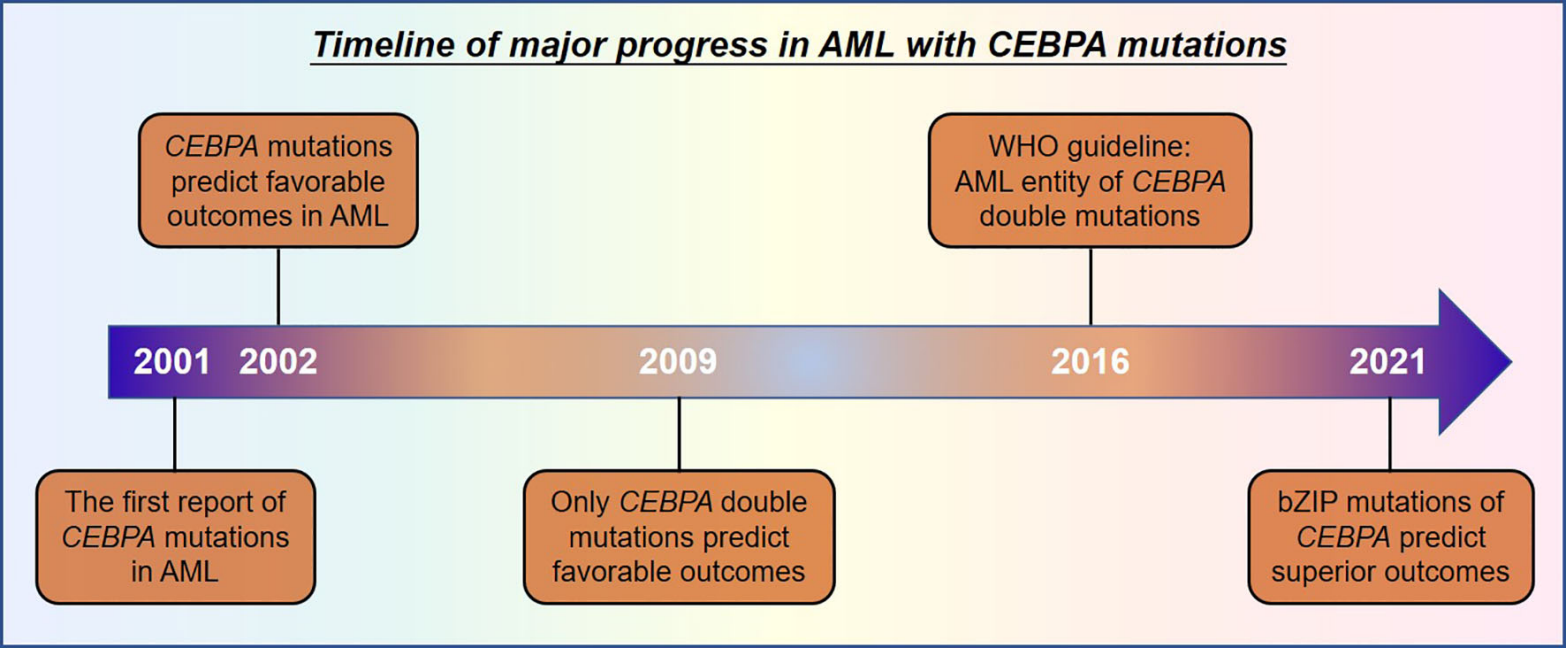
Major research progress of AML patients with *CEBPA* mutations.

The first study was published in 2001, reporting that *CEBPA* mutations were identified in 10 of 137 patients with AML, which was also the first report showing *CEBPA* mutations in human neoplasia (14). In the following year, the prognostic significance of *CEBPA* mutations was retrospectively analyzed in 135 AML (non-M3) patients. Fifteen patients were found harboring *CEBPA* mutations, which was demonstrated to be an independent favorable prognostic factor for long-term outcomes (15). In 2009, the prognostic significance of *CEBPA*^sm^ and *CEBPA*^dm^ was put forward by investigators from the Netherlands (13). Only patients with *CEBPA*^dm^ show a unique gene expression profile and favorable event-free survival (EFS) and overall survival (OS). However, both gene expression signature and outcomes were similar between patients with *CEBPA*^sm^ and wild-type *CEBPA* (13). Subsequently, a series of studies confirmed the favorable prognosis of AML with *CEBPA*^dm^, both in the whole patient cohort and those with normal karyotype (9, 10, 16, 17). Thus, AML with *CEBPA*^dm^ is recognized as a definite entity in “The 2016 revision to the World Health Organization classification of myeloid neoplasm and acute leukemia”, given its distinct biological and clinical characteristics (18). However, recent studies suggest that the classification of single and double mutations may not be sufficient to reflect the biological essence and clinic significance of such kind of AML. Recently, in a retrospective study including 4,708 adult patients with AML, the results showed that patients with *CEBPA*^dm^ and *CEBPA*^sm^ affecting bZIP (*CEBPA*^smbZIP^) shared similar gene expression profiles and clinical features, including younger age, higher leukocytes at diagnosis, and improved survival compared to those with *CEBPA*^sm^ affecting TAD (*CEBPA*^smTAD^). Further analysis revealed that the clinical and molecular characteristics and favorable outcomes were confined to patients carrying in-frame mutations in bZIP, regardless of single or double mutations, in terms of superior complete remission (CR) rates and long-term survival (19). The favorable prognosis of *CEBPA*^smbZIP^ was also observed in another independent patient cohort of 1,028 AML patients, and presence of *CEBPA*^smbZIP^ was a strong indicator of a higher chance to achieve CR, better survival, and lower risk of relapse (20). These studies may challenge the current concept of *CEBPA* mutations in diagnosis and treatment of patients with AML. New subsets of AML with bZIP or non-bZIP mutations of *CEBPA* may be recognized rather than single and double mutations. Moreover, the prognostic and therapeutic implications of AML with *CEBPA*^smbZIP^ may be similar to those with *CEBPA*^dm^. The major research progress of *CEBPA* mutated AML in the last two decades was summarized in **Figure 2**.

## Heterogeneities of AML With *CEBPA*^dm^

Although AML patients with *CEBPA*^dm^ show favorable outcomes, relapse after treatment is inevitable in many patients. Therefore, the heterogeneities of AML with *CEBPA*^dm^ have been noticed and discussed by our team and other investigators (2–6). Here, we divide these heterogeneities into two major categories, namely, genetic and treatment response heterogeneities.

### Genetic Heterogeneity

Mutations in transcription factor GATA2 were one of the most common molecular alterations in AML patients with *CEBPA*^dm^. In the preliminary study, whole exome sequencing was performed with five patients with *CEBPA*^dm^ and *GATA2* zinc finger 1 (ZF1) mutations were identified in two patients (21). The authors also found that the frequency of *GATA2* ZF1 mutations was 39.4% in AML patients with *CEBPA*^dm^, which tended to be a favorable indicator (21). Thereafter, several studies evaluated the prognostic significance of *GATA2* mutations in patients with *CEBPA*^dm^ (22–26) (summarized in **Table 1**). However, controversial results were found in those reports. Notably, high co-occurrence of other genetic mutations, such as *FLT3*-ITD in patients with wide-type *GATA2*, may produce unfavorable impact on the survival compared to those with mutated *GATA2*.

**Table 1:** Frequencies and clinical significance of *GATA2* mutations in AML with *CEBPA*^dm^.

Studies	Frequencies	ED	CR	EFS	OS
Fasan et al. (22)	18.3% (9/98)	NA	NA	Fav tendency	Fav
Grossmann et al. (24)	21.0% (20/95)	NA	NA	Fav tendency	Fav
Green et al. (23)	27.3% (15/55)	NS	NS	NA	NS
Marceau-Renaut et al. (25)	28.7% (25/87)	NA	NA	NA	NS
Theis et al. (26)	31.9% (36/113)	NS	NS	NS	NS

ED, early death; CR, complete remission; EFS, event-free survival; OS, overall survival; Fav, favorable; NA, not available; NS, not significant.

CSF3R is the receptor of granulocyte-colony stimulating factor (G-CSF), which functions through activation of the JAK/STAT signaling pathway. High occurrence of *CSF3R* mutations in AML patients with *CEBPA*^dm^ was first identified by RNA-sequencing in four of 14 patients, and all were T681I mutations (27). Meanwhile, high-frequency recurrent mutations in *CSF3R* were found with TARGET dataset of pediatric AML patients with *CEBPA* mutations (28). For the first time, we demonstrated that *CSF3R* mutations were associated with inferior survival in patients with AML with *CEBPA*^dm^ (5). Interestingly, *CSF3R* mutations were included in two recent studies as a parameter for prognostic nomograph models (29, 30). Thus, a high degree of overlap between *CSF3R* and *CEBPA* mutations may facilitate an in-depth understanding of the role of *CSF3R* in the pathogenesis and prognosis of AML patients with *CEBPA*^dm^, and development of new targeted therapy, which will be discussed in a subsequent section.

Other mutated genes, such as *TET*2 and *WT1*, were reported to be negative indicators for the prognoses of AML patients with *CEBPA*^dm^ (2, 3, 6, 24). Further studies are still needed to confirm these conclusions due to limited studies and relatively small numbers of patients with mutations. One effective way to solve the issue of small patient size is to combine patients with mutations according to gene family or pathways. Mutations of tyrosine kinase genes, including *FLT3*, *CSF3R*, *KIT*, and *JAK*3, confer adverse prognosis (31). Two genetic subgroups were defined by the presence (positive; pos) or absence (negative; neg) of mutations in chromatin/DNA modifiers (C), cohesin complex (C), and splicing (S) genes: CCSpos and CCSneg, respectively. Only patients with *CEBPA*^dm^ with CCSneg had distinct genetic and clinical features and favorable outcomes compared to those with *CEBPA*^sm^ (3). Interestingly, most patients (20/25, 80%) in the CCSpos group were defined by *TET2* mutations in this study, which may reflect the unfavorable impact of *TET2* mutations on the survival of AML with *CEBPA*^dm^.

### Treatment Response Heterogeneity

Although CR rate after induction chemotherapy is very high in patients with *CEBPA*^dm^, a substantial proportion of the patients (30%–50%) will relapse consolidated with chemotherapeutic agents only (2, 32, 33), which suggests the heterogeneity of treatment responses of these patients. Measurable residual disease (MRD) status is a very important indicator for treatment responses and prognosis in patients with AML, which is also a potential biomarker for prognostic restratification of AML with *CEBPA*^dm^. In the preliminary single-center study, patients with *CEBPA*^dm^ were divided into MRD high-risk (positive after two consolidation cycles and/or negative status loss at any time) and low-risk (persistent negative) groups based on MRD status during consolidation therapy (33). As expected, MRD risk groups were the only independent risk factor for relapse and long-term survival in multivariate analysis (33). Subsequently, we conducted a multicenter retrospective study that also confirmed the previous findings that only MRD low risk associated with low recurrence rate and superior outcomes in multivariate analysis (unpublished). Therefore, MDR status may be a potential indicator to be considered for treatment choice in patients with *CEBPA*^dm^. However, it should be noted that these two categories of heterogeneities may not be separated absolutely, because we notice that patients with high-risk genetic mutations, such as mutated *CSF3R*, had a significantly higher rate of positive MRD than those with wide-type *CSF3R* after consolidation therapy (82.0% vs. 56.25%, respectively).

## Specific Subtypes of AML With *CEBPA* Mutations

### Pediatric Patients With *CEBPA* Mutations

AML in adults and children may show different biological behaviors, treatment responses, or prognoses. In 2005, the first study reported that the frequency of *CEBPA* mutations was 6.19% (7/113) in pediatric patients with AML, including two with single and five with double mutations. Four of the seven patients had cooperating mutations with *FLT3*-ITD or *NRAS* mutations (34). The prevalence and prognostic significance of *CEBPA* mutations were evaluated in 847 children with AML from 3 consecutive clinical trials. *CEBPA* mutations were detected in 38 patients (4.49%), with 31 cases harboring double mutations. Patients with *CEBPA* mutations had significantly improved EFS and OS, and lower cumulative incidence rate of relapse compared to those with wide-type *CEBPA* (35). Single (*n* = 7) or double (*n* = 31) mutations had no significant impact on the prognosis of these patients (35), which may be due to the small size of patients in each arm. In another study from Japan, a high frequency of *CEBPA* mutations (14.92%, 47/315) was observed, and *CEBPA*^dm^ is an independent favorable prognostic risk factor in pediatric AML patients in multivariate analysis in the total patient cohort (36). Hence, the favorable prognostic significance of *CEBPA* mutations could also be confirmed in pediatric patients with AML.

### Familial AML With *CEBPA* Mutations

As early as 1978, a large familial aggregation leukemia was reported, and 13 individuals over four generations of a family comprising 293 members were diagnosed (37). After screening of genetic markers, karyotypes, and virus infections, the authors postulated that such aggregation of leukemia cases likely resulted from undefined genetic, probably polygenic, predisposition, in association with the activity of leukemogenic factors (37). However, the riddle was solved 30 years later. In 2010, a report based on one member of this family (III-45) was diagnosed as AML carrying a single heterozygous base pair deletion of the N-terminal (c.68delC) in somatic sample and a probable acquired three-base pair duplication (c.937_939dupAAG) in the C-terminal of *CEBPA* in a proportion of peripheral blood cells, indicating familial AML with *CEBPA* mutations (38). Small cases of familial AML with *CEBPA* mutations were also reported by other studies (23, 39). In 2015, the first study exploring the disease evolution and outcomes of familial AML with germline *CEBPA* mutations was reported, and 24 members from 10 *CEBPA*-mutated families were enrolled (40). Germline *CEBPA* mutations clustered within the N-terminal and acquired mutations preferentially targeting the C-terminal in diagnostic leukemia samples. AML patients with germline *CEBPA* mutations showed absence of diagnostic *CEBPA* mutations in relapse (40) and younger age than those with sporadic *CEBPA* mutations (41). Furthermore, patients with familial *CEBPA* mutations showed a favorable long-term outcome with 10-year OS of 67% (40). Although familial AML with *CEBPA* mutations is a rare disease, these studies discovered the unique biological behaviors and favorable prognosis of these patients.

## Treatment Strategies for Patients With *CEBPA* Mutations

High CR rates of *de novo* (~90%) and relapsed (~80%) AML patients with *CEBPA*^dm^ induced by chemotherapy indicate that this subtype of AML is highly sensitive to chemotherapeutic agents (42). Furthermore, with the insight into the pathogenesis and clinical features of *CEBPA* mutated AML in recent years, therefore, it is necessary to reconsider the treatment choice for these patients. A comparison between hematopoietic stem cell transplantation (HSCT) and chemotherapy was performed with 124 patients with *CEBPA*^dm^ in CR1. Thirty-two patients were treated with allogeneic HSCT (allo-HSCT), 20 with autologous HSCT (auto-HSCT), and the remaining 72 received chemotherapy. Although patients consolidated with chemotherapy showed significantly higher relapse rates compared to those in both auto-HSCT and allo-HSCT groups, such advantage did not translate into survival benefit for HSCT. Furthermore, there is no significant difference between patients in auto-HSCT and allo-HSCT groups in terms of relapse-free survival and OS (32). Relapsed patients still have a favorable outcome after reinduction followed by allo-HSCT with a 3-year OS of 46% (32). Allo-HSCT and chemotherapy were also compared in AML patients with *CEBPA*^dm^ in other studies. Allo-HSCT (*n* = 25) resulted in significantly lower incidence rate of relapse than chemotherapy (*n* = 24), but OS was similar between those two groups (43). Another study favored chemotherapy, not allo-HSCT, for patients with *CEBPA*^dm^ (44). In a recent study, *CEBPA*^dm^ AML patients were divided into low- and high-risk groups according to a nomograph model that was constructed with high white blood cell counts, DNA methylation related gene, *CSF3R*, and *KMT2A* mutations. Allo-HSCT was superior to chemotherapy and was only observed in high-risk, but not low-risk subgroups (29). Collectively, these results suggest that the majority of studies showed that allo-HSCT was not superior to chemotherapy or auto-HSCT in AML with *CEBPA*^dm^. Nevertheless, certain AML patients with *CEBPA*^dm^ may benefit from allo-HSCT, but further study is needed to explore and validate.

With the emerging research advances, other potential targets that are reported in AML with *CEBPA*^dm^ may be used for treatment. AML with *CEBPA*^dm^ showed a low genetic expression signature, and reactivation of these low expressed genes promoted granulocytic differentiation of primary samples by histone deacetylase inhibitors that may be a candidate for treatment (45). High frequency of *CSF3R* mutations was discovered in AML with *CEBPA*^dm^, which was sensitive to JAK inhibition; furthermore, AML patients with *CEBPA*^dm^ with special gene expression prolife without *CSF3R* mutations were uniformly sensitive to JAK inhibitors as well, which suggests the possibility of using JAK inhibitors in those patients (27). In addition, a combination of inhibitors of JAK signaling pathway and lysine-specific demethylase 1 is effectively capable of controlling the growth of *CSF3R*/*CEBPA* mutant leukemia *in vivo* (46). The interaction between MLL histone-methyltransferase complex with CEBPα p30 plays a critical role in leukemogenesis of *CEBPA* mutated AML, while MLL inhibition impairs proliferation and restores myeloid differentiation in AML cells with *CEBPA* mutations (47). As both histone deacetylase inhibitor Chidamide and JAK inhibitor Ruxolitinib have been used in clinic, integration of these inhibitors with chemotherapy or HSCT may possibly improve the prognosis of AML with *CEBPA* mutations.

## Conclusion and Future Directions

**Figure 3:**
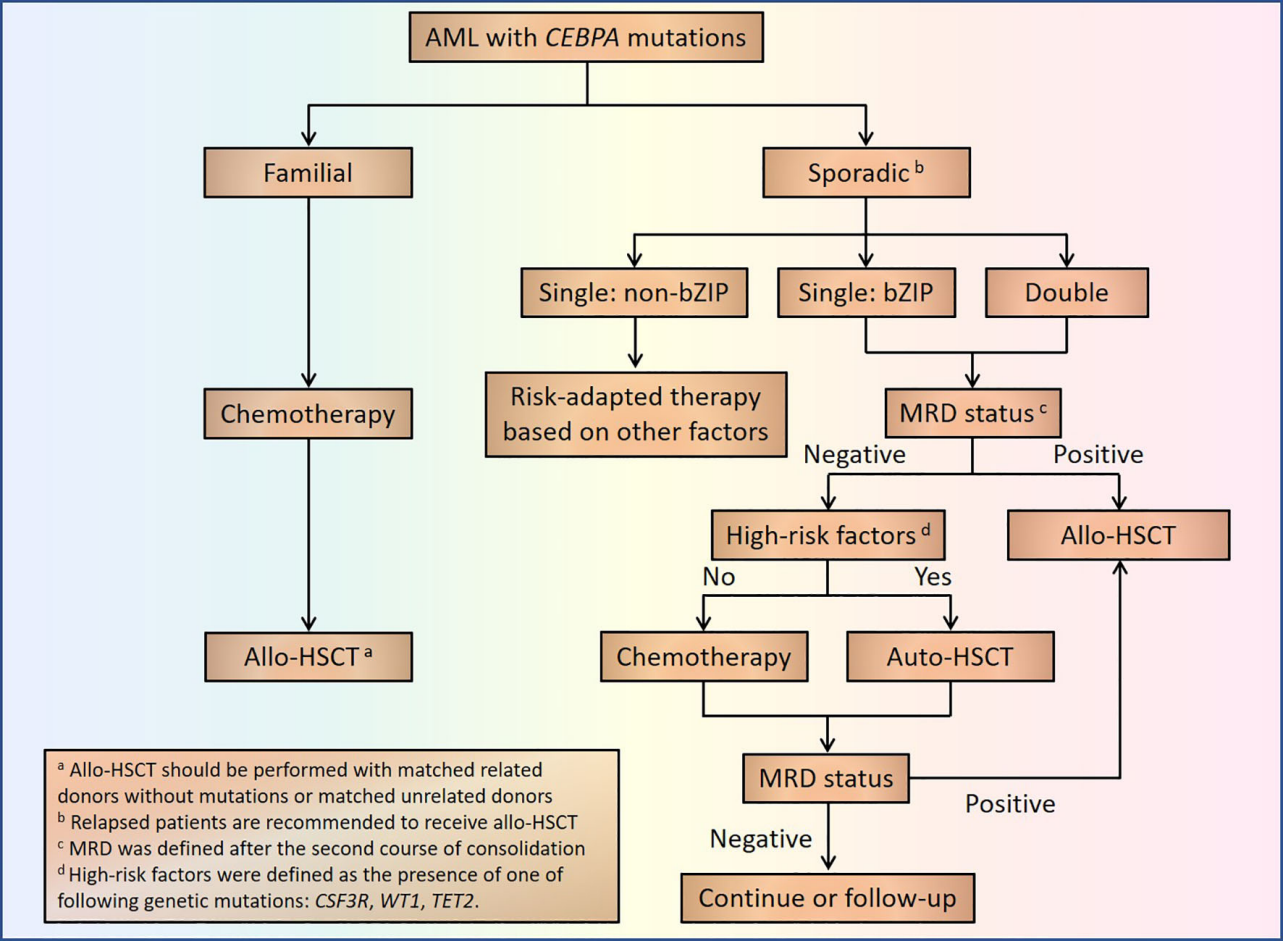
Treatment flowchart of AML patients with *CEBPA* mutations.

From what was discussed above, we could see that AML patients with *CEBPA*^dm^ are sensitive to chemotherapy, which suggests a critical role of chemotherapy and auto-HSCT in the treatment of those patients. Although some genetic mutations are associated with high risk of relapse (*CSF3R*, *WT1*, and *TET2*; high-risk factors) in AML with *CEBPA*^dm^, the total frequency of those mutations is higher than the recurrence rate of *CEBPA*^dm^ patients consolidated with auto-HSCT, which indicates that patients with those high-risk factors may also benefit from auto-HSCT. Furthermore, as the majority of patients with *CEBPA*^dm^ carry mutations in bZIP, it will result in limited significance to divide *CEBPA*^dm^ into those with or without bZIP mutations. However, recent research indicates that *CEBPA*^sm^ located in bZIP showed similar clinical features and prognosis to those with *CEBPA*^dm^. Therefore, we propose that AML patients with sporadic *CEBPA* mutations should be divided into *CEBPA*^smnon-bZIP^, *CEBPA*^smbZIP^, and *CEBPA*^dm^ for further treatment. For those with *CEBPA*^smbZIP^ and *CEBPA*^dm^, they should be treated according to MRD status and genetic high-risk factors for choosing chemotherapy, auto-HSCT, or allo-HSCT as we presented in **Figure 3**. Optimization of prognostic evaluation and treatment choice for AML patients with *CEBPA* mutations by MRD status during treatment here may suggest that an integrated prognostic system should be established with both pre-treatment (cytogenetic and genetic alterations) and post-treatment (MRD status) parameters, in order to direct choosing treatment strategies post remission. As to those with familial AML with *CEBPA* mutations, favorable outcomes could be achieved by chemotherapy, and those with refractory or relapse disease should receive allo-HSCT to eliminate the germline mutations with related donors without mutations or unrelated donors (**Figure 3**).

More beneficial evidence that *CEBPA* bZIP mutations may define a subset of AML is still anticipated, especially in the settings of different populations or treatment plans. Some investigators suggested the classification of *CEBPA* mutated AML as *CEBPA* with in-frame bZIP mutations and those without. However, two points must be mentioned. First, the frequency of frame-shift bZIP mutations in *CEBPA*^dm^ is very low in AML in some patient cohorts; it is only 4.38% (6/137) in patients from our center. Second, a comparison between in-frame and frame-shift bZIP mutations of *CEBPA* is still needed. Furthermore, whether such phenomenon could be observed in pediatric AML patients needs further exploration. Although AML with *CEBPA*^dm^ is sensitive to chemotherapy, evidence of auto-HSCT is limited, which may be helpful to prevent disease relapse in some patients because auto-HSCT is more intensive than chemotherapy alone. Finally, with the discovery of new potential targets or development and application of new drugs in the treatment of those patients, the prognoses of *CEBPA* mutated AML may be further improved, which may challenge the diagnosis and treatment dogma of the current concept.

## Author Contributions

LS wrote the manuscript. LS, Y-YS, Z-YL, and S-JG collected, analyzed, and summarized the data. LS, Y-YS, Z-YL, and S-JG conceptualized this review. LS and S-JG revised the review. All authors contributed to the article and approved the submitted version.

## Funding

This work was supported by grants from NSFC (81900174) and the Clinical Research Foundation of First Hospital of Jilin University (No. LCFYJJ2017005).

## Conflict of Interest

The authors declare that the research was conducted in the absence of any commercial or financial relationships that could be construed as a potential conflict of interest.

## Publisher’s Note

All claims expressed in this article are solely those of the authors and do not necessarily represent those of their affiliated organizations, or those of the publisher, the editors and the reviewers. Any product that may be evaluated in this article, or claim that may be made by its manufacturer, is not guaranteed or endorsed by the publisher.
